# Altered Local Spatiotemporal Consistency of Resting-State BOLD Signals in Patients with Generalized Tonic-Clonic Seizures

**DOI:** 10.3389/fncom.2017.00090

**Published:** 2017-09-29

**Authors:** Shuai Ma, Sisi Jiang, Rui Peng, Qiong Zhu, Hongbin Sun, Jianfu Li, Xiaoyan Jia, Ilan Goldberg, Liang Yu, Cheng Luo

**Affiliations:** ^1^Neurology Department, Sichuan Provincial People's Hospital, The Affiliated Hospital of University of Electronic Science and Technology of China, Chengdu, China; ^2^Key Laboratory for NeuroInformation of Ministry of Education, Center for Information in Medicine, High-Field Magnetic Resonance Brain Imaging Key Laboratory of Sichuan Province, School of Life Science and Technology, University of Electronic Science and Technology of China, Chengdu, China; ^3^Neurology Department, Wolfson Medical Center, Holon, Israel

**Keywords:** generalized tonic-clonic seizures, epilepsy, spatiotemporal consistency, spontaneous fluctuation, resting-state fMRI

## Abstract

The purpose of this study was to evaluate the spatiotemporal Consistency of spontaneous activities in local brain regions in patients with generalized tonic-clonic seizures (GTCS). The resting-state fMRI data were acquired from nineteen patients with GTCS and twenty-two matched healthy subjects. FOur-dimensional (spatiotemporal) Consistency of local neural Activities (FOCA) metric was used to analyze the spontaneous activity in whole brain. The FOCA difference between two groups were detected using a two sample *t*-test analysis. Correlations between the FOCA values and features of seizures were analyzed. The findings of this study showed that patients had significantly increased FOCA in motor-related cortex regions, including bilateral supplementary motor area, paracentral lobule, precentral gyrus and left basal ganglia, as well as a substantial reduction of FOCA in regions of default mode network (DMN) and parietal lobe. In addition, several brain regions in DMN demonstrated more reduction with longer duration of epilepsy and later onset age, and the motor-related regions showed higher FOCA value in accompany with later onset age. These findings implicated the abnormality of motor-related cortical network in GTCS which were associated with the genesis and propagation of epileptiform activity. And the decreased FOCA in DMN might reflect the intrinsic disturbance of brain activity. Moreover, our study supported that the FOCA might be potential tool to investigate local brain spontaneous activity related with the epileptic activity, and to provide important insights into understanding the underlying pathophysiological mechanisms of GTCS.

## Introduction

Epilepsy is recognized as a neuropsychic disease with hyper-synchronous neuronal activity (Schevon et al., 2007). Idiopathic generalized epilepsy (IGE) is characterized by the widespread generalized spike-and-waves (GSWDs) without detectable focal anatomical brain abnormalities (Nordli, 2005). Meanwhile, some cognitive impairments have been reported in the IGE patients, such as attention, memory, and language dysfunctions (Gotman et al., 2005). Generalized tonic-clonic seizures (GTCS) is the most common syndrome of IGE, which showed serious clinical manifestations with seizures that result mental impairment and high mortality associated with epilepsy (Danielson et al., 2011).

Although substantial efforts have been made in the past decade, the pathophysiological mechanisms of GTCS remain largely unclear. Recent advances in neuroimaging techniques have provided efficient and noninvasive ways for better understanding of GTCS. Blood oxygenation level-dependent (BOLD) fMRI has been recognized as an effective noninvasive technique to investigate epilepsy (Luo et al., 2014; Cao et al., 2017; Ji et al., 2017). Seizure dynamics of epilepsy have been studied using the epileptor model (Guo et al., 2017). Simultaneous EEG and functional MRI (EEG-fMRI) has been used to study the brain activity in patients with IGE (Gotman et al., 2005; Li et al., 2009). Growing evidence has demonstrated morphological changes and altered structural connectivity in epilepsy (Xue et al., 2014; Gong et al., 2017). The investigation of brain networks in epilepsy has become a key concept to deeply understand the disease. The diffusion tensor imaging (DTI) was used to study the networks of epilepsy, and it helped reveal potential structure basis of the functional integrations among subcortical and cortical areas (Ji et al., 2014). Resting-state fMRI is a powerful tool to study the mechanisms of functional alterations in various neurological disorders, such as epilepsy (Yang et al., 2014; Li et al., 2015), chronic obstructive pulmonary disease (Yu et al., 2016), schizophrenia (Chen et al., 2015, 2017; Duan et al., 2015) and Alzheimer's disease (Wu et al., 2011). A recent fMRI study has demonstrated that the resting state networks (RSNs) are impaired in GTCS (Zhong et al., 2011), and many recent fMRI studies have found altered brain activity of default mode network (DMN) in the patients with GTCS (Song et al., 2011). In addition, the abnormal brain activity in the DMN might be correlated to the disturbance of consciousness during GTCS seizures (Cavanna and Monaco, 2009).

In this study, we attempt to investigate the intrinsic resting-state brain activity of GTCS patients. Four-dimensional (spatiotemporal) Consistency of local neural Activities (FOCA) is a new method proved to be effective in the investigation of temporal and spatial information of the local region (Dong et al., 2015). FOCA focus on both temporal homogeneity and regional stability, which may be considered to reflect local functional states powerfully (Dong et al., 2015). From a computational point of view, compared with the ReHo metric, FOCA metric is stricter because it combines the temporal and spatial information to detect the consistency of brain activity at the voxel level. Since IGE has been recognized as a neurological disorder which implicates widespread local brain regions, we presume that the FOCA metric possesses higher efficacy to detect the regions with aberrant spontaneous activity and provides more reliable results to reveal epileptic brain spontaneous activity in patients with GTCS. The FOCA metric has been implied to frontal lobe epilepsy, which revealed abnormal FOCA in DMN, frontal regions, basal ganglia and cerebellum (Dong et al., 2016). Besides, the FOCA has been used in the study of schizophrenia to investigate the local consistency (Chen et al., 2017). Taken together, the FOCA features would have potential to reveal the functional alterations of local spontaneous activity in patients with GTCS. In addition, it has been widely accepted that epileptic seizures may be caused by abnormal neuronal synchronization (Jiruska et al., 2013). In this study, we aim to investigate local spatiotemporal consistency of spontaneous activity in GTCS patients using resting-state fMRI, which may provide meaningful information for understanding the illness mechanism of GTCS.

## Materials and methods

### Subjects

Nineteen patients (mean age: 22.9 ± 8.8 years; mean years of illness duration: 6.2± 6.2, 9 females) with GTCS were recruited in the Center for Information in Medicine, University of Electronic Science and Technology of China. All patients recruited were diagnosed as GTCS only relied on the clinical and seizure semiology information in line with the International League Against Epilepsy (ILAE) guidelines (Engel, 2001). Sixteen patients took antiepileptic drugs, the remained were diagnosed newly and did not receive any treatment. All the routine brain neuroimaging, such as CT, showed no structural abnormalities; scalp EEG demonstrated GSWDs. Twenty-two sex- and age-matched healthy controls were recruited for the control group (mean age: 26.1 ± 8.7 years, 12 female). All the controls showed normal neurologic examination and normal MRI. This study was approved by the ethical committee of the University of Electronic Science and Technology of China according to the standards of the Declaration of Helsinki. Written informed consent was obtained from all of the subjects.

### Data acquisition

All subjects underwent MRI scanning in a 3T GE scanner with an eight-channel-phased array head coil (MR750; GE Discovery, Milwaukee, WI) in the MRI research center of University of Electronic Science and Technology of China. An echo-planar imaging sequence was utilized to collect resting-state functional data, with the following parameters: echo time (*TE*) = 30 ms, repetition time (*TR*) = 2,000 ms, flip angle (*FA*) = 90, matrix = 64 × 64, field of view (*FOV*) = 24 × 24 cm^2^, and slice thickness = 4 mm with 0.4 mm gap, and 255 volumes in each run. A 3-dimensional fast spoiled gradient echo (T1-3D FSPGR) sequence was used to acquire axial anatomical T1-weighted images with 152 slices per volume. The acquisition was performed with *TE* = 1.984 ms, *TR* = 6.008 ms, *FA* = 90, matrix = 256 × 256, *FOV* = 25.6 × 25.6 cm^2^, slice thickness = 1 mm (no gap). During resting-state fMRI scanning, During the examination, all subjects were instructed to be “relaxed, eyes closed” and kept awake and not to think of anything in particular.

### Data preprocessing

The SPM8 software package (statistical parametric mapping available at: http://www.fil.ion.ucl.ac.uk/spm) was used to preprocess fMRI data. The first five volumes of each run were discarded to remove the T1 saturation effects. The remaining 250 volumes were slice-timing corrected and realigned. We excluded subject whose head motion exceeded 1.5 mm or/and 1.5°. The realigned images were spatially normalized to the Montreal Neurological Institute (MNI) template and resliced with voxel size of 3 × 3 × 3 mm. No temporal filtering was performed in this processing in consideration of the following analyses in full frequency band. Finally, the unsmoothed resting-state fMRI data regressed out six head motion parameters, individual mean white matter, and cerebrospinal fluid signals.

### FOCA analysis

The FOCA calculation was performed using a neuroscience information toolbox (NIT, v1.1, RRID:SCR_014501 http://www.neuro.uestc.edu.cn/NIT.html). FOCA have been demonstrated to assess the spatiotemporal consistency of local spontaneous activity. The value of FOCA is the absolute value of the product of two types of correlation. One is temporal correlation, which reflects the across cross-correlation coefficients of adjacent 27 voxels for a given voxel. The other is spatial correlation, which evaluates the correlations between local spatial distributions in the neighboring time points. Dong et al. have made detailed demonstrations of FOCA (Dong et al., 2015). The FOCA values were normalized by dividing the mean value of the whole brain. Besides, the mean FOCA value of whole brain was subtracted from these normalized maps, making zero-mean FOCA maps for further statistical analysis. To evaluate the local spontaneous brain activity in both groups, one sample *t*-tests were performed in the FOCA map after smoothing the FOCA map with Gaussian kennel function (8 mm full-width at half maximum). Then, a two sample *t*-test was utilized to study the difference between GTCS and the control group. We further transformed these *t*-values into *z*-values to display the statistical results. The significance was set at *P* < 0.05 with FDR correction for multi-compares and the cluster correction. The average normalized FOCA values were extracted from the regions which demonstrated significant alterations between groups. The partial correlation coefficients were calculated between the FOCA values extracted and disease duration and age of onset, with controlling the effect of gender.

## Results

No excessive head motion was observed in our subjects.

### Within-group and between-group FOCA values

**Figure 1:**
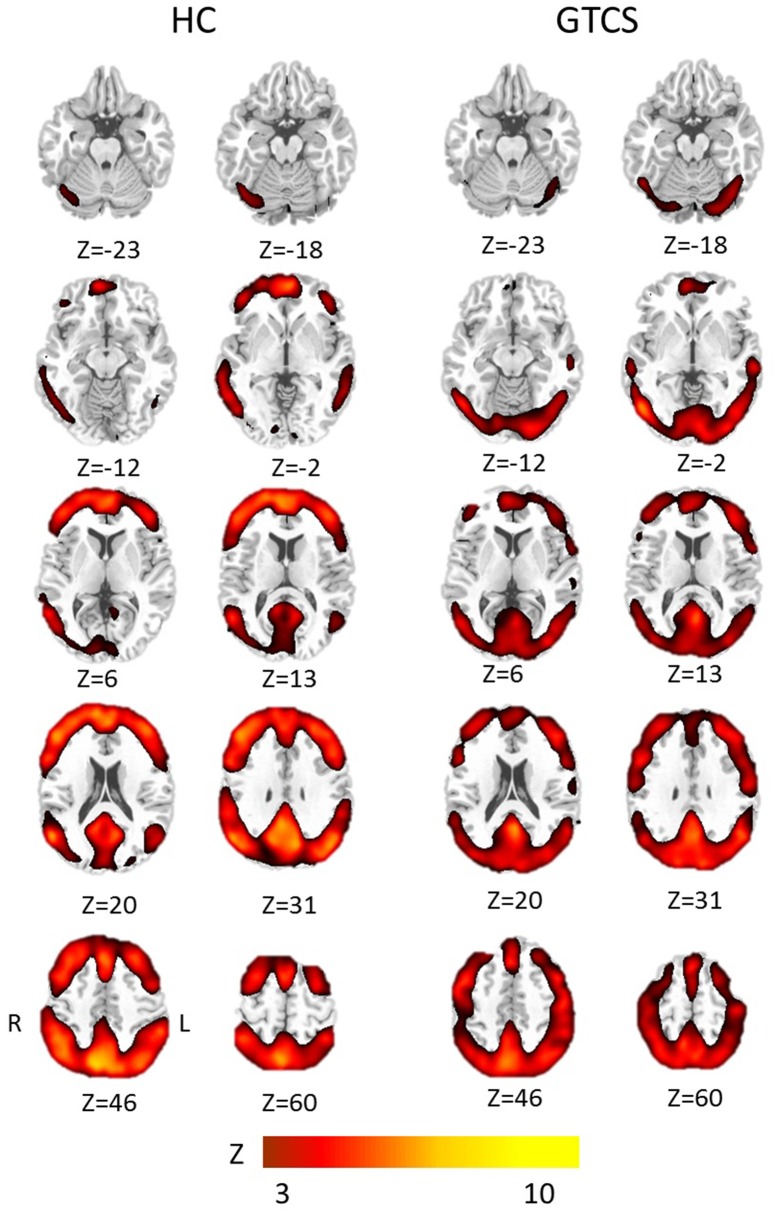
The group results of high FOCA values in patients with GTCS and healthy controls. Significant regions with a threshold *Z* > 3 (*p* < 0.05, FDR corrected) were shown in each group, respectively. HC, Healthy control group; R, right; L, left.

The results of the one-sample *t*-test in both groups were shown in Figure 1, respectively. The voxels with high FOCA value was found in the major regions of the DMN, such as the medial frontal lobe, posterior cingulate cortex, and other regions including the frontal and parietal cortex, visual cortex, and cerebellum.

**Figure 2:**
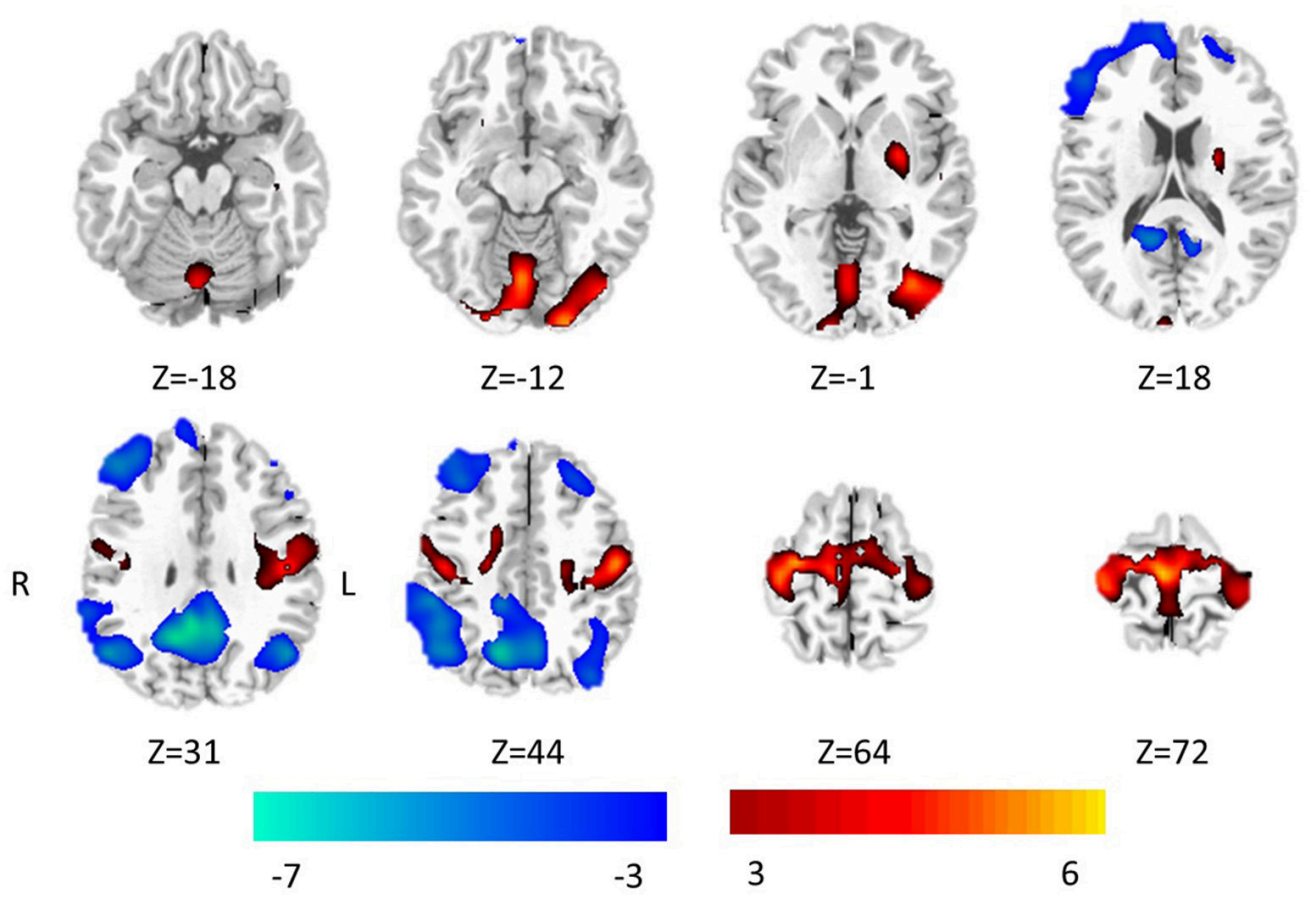
Statistic Z-map showing the FOCA difference between the GTCS group and the healthy control (*p* < 0.05 with FDR correction). The Z values were shown with hot color for positive values (GTCS > healthy controls) and cool colors for negative values (GTCS < healthy controls).

The significant differences of FOCA between two groups (*P* < 0.05, FDR-corrected) were demonstrated in Figure 2, and detailed information of clusters with difference was recorded in Table 1. Compared with the healthy controls, GTCS patients showed significantly increased FOCA values at bilateral supplementary motor area (SMA), and paracentral lobule, left inferior occipital gyrus, right lingual gyrus, bilateral postcentral, precentral left putamen and vermis. Significantly decreased FOCA values were also shown in bilateral precuneus, bilateral angular gyrus, bilateral middle frontal cortex, right inferior frontal cortex and right medial prefrontal cortex.

**Table 1:** Brain regions showing significantly different FOCA in patients with GTCS.

**Brain region**	**MNI coordinates**			**BA**	***T*-value**	**Voxel number**
	**X**	**Y**	**Z**			
**GTCS > HC**						
Supp_Motor_Area_B	9	−20	64	4	5.06	1103
Precental_B	40	−25	62	4	5.1	
Postcentral_B	45	−20	38	3	4.23	
Paracentral_lobule_B	3	−22	68	4	5.32	
Putamen_L	−28	−5	−4	-	4.38	63
Occipital_Mid_L	−33	−76	0	19	4.54	275
Occipital_Inf_L	−41	−82	−10	19	4.95	
Lingual_R	15	−97	−10	18	4.88	146
Vermis_6	2	−74	−10	-	5.11	
Lingual_L	−22	100	−13	18	4.94	
Cerebellum_Crus2_R	7	−79	−40	-	3.51	
Cerebellum_6_L	−32	−41	−37	-	3.68	53
**GTCS < HC**						
Frontal_Mid_R	43	38	32	46	4.59	630
Frontal_Sup_R	23	42	44	9	3.72	
Frontal_Sup_Medial_R	8	59	18	10	3.92	
Frontal_Inf_Tri_R	53	36	14	45	3.97	
Angular_L	−50	−65	27	39	4.78	203
Parietal_Inf_L	−35	−78	42	7	4.25	
Occipital_Mid_L	−42	−75	32	39	4.35	
Precuneus_B	11	−62	32	-	7.03	481
Angular_R	47	−65	32	39	5.32	
Cinglum_Post_B	3	−53	32	23	6.27	
Parietal_Inf_R	49	−50	40	40	4.76	
Frontal_Mid_L	28	30	44	9	4.16	153
Frontal_Sup_L	23	32	45	9	4.25	
Cerebelum_9_R	10	−53	−48	-	3.44	

*MNI, Montreal Neurological Institute; BA, Brodmann area; L, left; R, right; B, bilateral*.

### Correlation analyses

**Figure 3:**
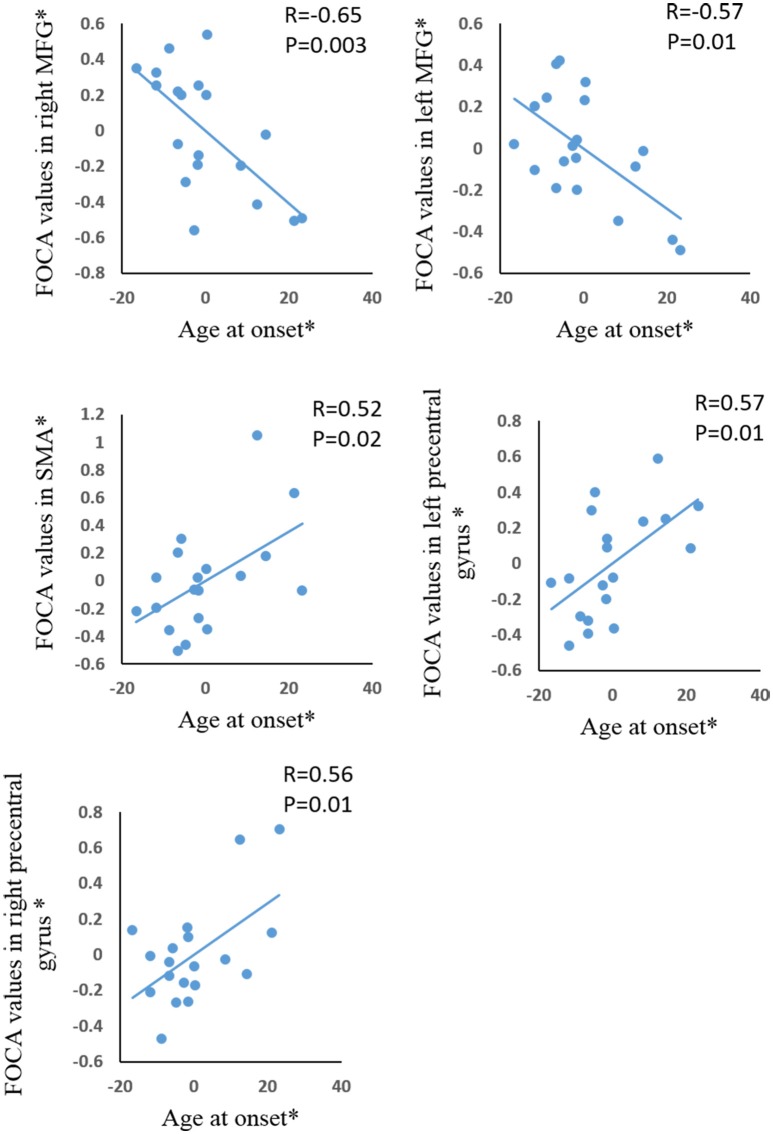
The correlation between age at onset and FOCA values in the brain regions which showed significant group difference. ^*^The coordinate value implicates the residuals after controlling for the influence of the gender (linear regression with covariates including gender).

**Figure 4:**
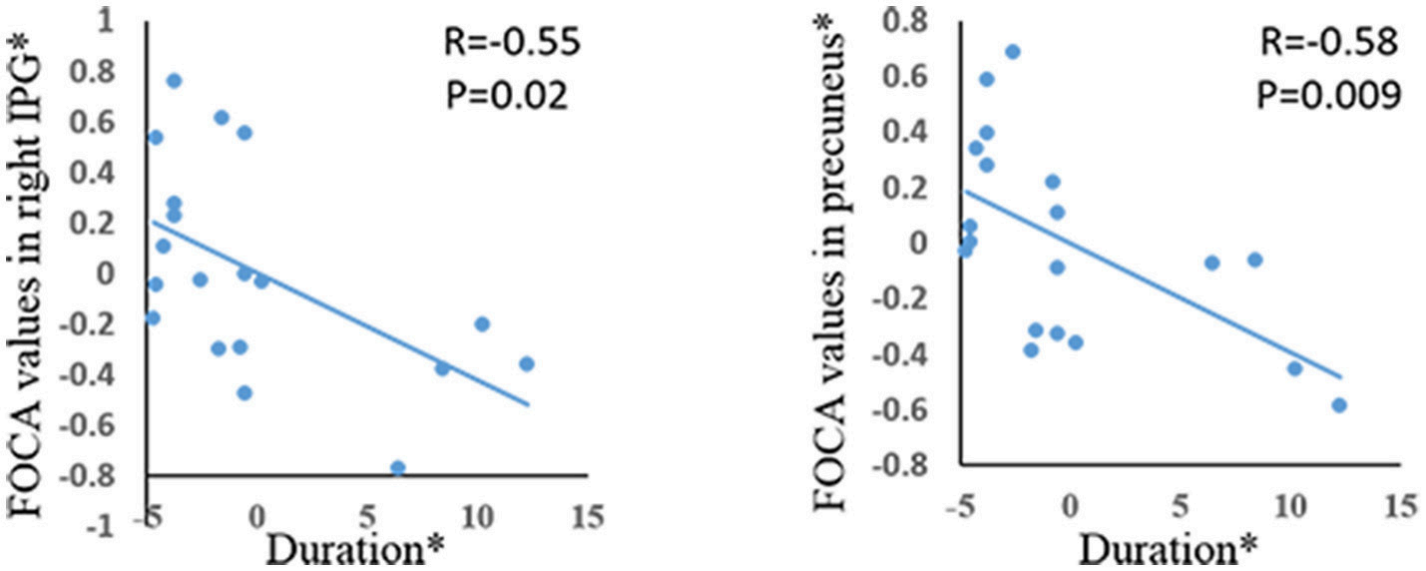
The correlation between duration of epilepsy and FOCA values in the brain regions which showed significant group difference. ^*^The coordinate value implicates the residuals after controlling for the influence of the gender (linear regression with covariates including gender).

Significant positive correlations were demonstrated between age of onset and FOCA value in bilateral SMA and bilateral paracentral lobule (Figure 3). The bilateral medial frontal lobe cortex showed negative correlation with the onset age of epilepsy (Figure 3). In addition, the precuneus and right inferior parietal gyrus negatively related with illness duration (Figure 4).

## Discussion

To our knowledge, this is the first study to investigate the alterations of spatiotemporally spontaneous activity in GTCS patients, using the FOCA feature in resting-state fMRI. The patients with GTCS demonstrated increased FOCA in motor related regions (SMA and pre-motor), basal ganglia and cerebellum, and decreased FOCA in DMN when compared with controls. In addition, several brain regions in DMN demonstrated a greater reduction with a longer duration of epilepsy and a later onset age, and the motor-related regions showed enhanced FOCA value with a later onset age.

The brain activity has been widely studied using various metrics, in which the ReHo is a known measurement to investigate the synchronization of spontaneous BOLD oscillations within local brain regions (Zang et al., 2004). A study demonstrated the alteration of ReHo in patients with GTCS, which showed some consistent findings in our work, such as decreased synchronization in DMN and increased in sensorimotor areas (Zhong et al., 2011). Meanwhile, our previous study utilizing ReHo demonstrated altered local spontaneous brain activity in JME (Jiang et al., 2016), which showed similar results to Zhong's. Compared with these previous studies, some different findings were shown in the present work. FOCA metric showed some specific findings in patients with epilepsy, which possess powerful potential to reveal the brain regions involved in disease. In addition, more concentrated intergroup difference areas were shown in the FOCA statistical maps.

In the present work, compared with healthy controls, GTCS patients demonstrated decreased FOCA values in DMN. It has been suggested that the activity of DMN represent the intrinsic function of human brain (Shulman et al., 1997; Raichle et al., 2001). Besides, some investigators demonstrated that the DMN plays a key role in processing internal mentation and external stimulation (Buckner et al., 2008).

It has been demonstrated that epileptic activity may interrupt the resting state and result in the deactivation of the DMN (Luo et al., 2011; Song et al., 2011). Reduced regional synchronization in the posterior DMN regions has been demonstrated in juvenile myoclonic epilepsy, which is consistent with the observations in this work. It has been demonstrated that PCC showed reduced activation during interictal epileptic discharges and reduced cerebral blood flow in patients with GTCS (Joo et al., 2008), which provides evidence showing that the PCC might be related with the generation or propagation of epileptic activity. In this study, we also find significant alterations of FOCA in PCC. It has been found the changed functional.

Connectivity within the DMN of the GTCS patients (Ralchle and Snyder, 2007) and patients with temporal lobe epilepsy (Morgan et al., 2007), which might indicate possible reorganization of DMN. In addition, some researchers believe that the abnormal brain activity in the DMN might correlate with the complete impaired consciousness of the GTCS patients during seizures (Cavanna and Monaco, 2009). In the current study, we demonstrate the alterations of FOCA values in the DMN, which reveal functional alterations of local spontaneous activity. Combined with findings of previous studies, we infer the decreased FOCA in the precuneus in GTCS patients may be related with the effects of epileptic activity, which may contribute to the abnormalities of functional connectivity in DMN relating to the conscious disturbance of patients with GTCS. Furthermore, the negative correlation was observed between the FOCA in the precuneus and the duration of patients with GTCS. That means the injury of DMN increases gradually along with the development of disease. In addition, negative correlations were observed between the bilateral medial frontal gyrus and the onset of GTCS, which could be interpreted as the effect of neural plasticity.

In the present study, the increased FOCA values are mainly located in the SMA and pre-motor system. A notion has been proposed in previous study that the myoclonic jerks in IGE may be caused by the motor circuitry hyper-excitability (Vollmar et al., 2011). In addition, more and more structural and functional evidence demonstrates that the motor regions, such as SMA and precentral gyrus play an important roles in the propagation of generalized epileptic activity (Anderson and Hamandi, 2011; Caeyenberghs et al., 2015). Our findings showed the abnormal FOCA values in the motor-related brain regions in patients with GTCS, which positively correlated with the onset age of disease. Consistent with previous findings, we presume that the SMA might be a crucial node in the epileptic network and be associated with the motor abnormality of GTCS patients.

Compared with controls, patients with GTCS showed significantly increased FOCA values in the left basal ganglia (BG). The BG forms a very complex system of nuclei and pathways and may act as an integrated system. As other studies have shown, subcortical areas including putamen were connected with a wide range of brain regions, including the frontal lobe, parietal lobe, temporal lobe and cerebellum. The disruption of functional connectivity in basal ganglia networks was identified using independent component analysis (Luo et al., 2012). In addition, functional resting-state network studies have identified disturbed connectivity between the BG with large scale cortical networks (Rektor et al., 2013). Based on direct ictal recordings from the basal ganglia (Rektor et al., 2002) and anterior nucleus of thalamus (Rektor et al., 2016) via depth electrodes, the researchers have suggested an important role of subcortical structures in focal seizures. Increased studies focused on the role of the BG in epilepsy in terms of their prospective application as deep brain.

Stimulation targets for controlling seizures. For example, it has been proposed that the basal ganglia nuclei are structures controlling cortical seizure activity (Rektor et al., 2012). We suppose these alterations of spontaneous activity in BG in GTCS patients might be associated with epileptic activity. However, the roles of the basal ganglia in GTCS would be further studied in the future.

Furthermore, patients with GTCS showed the enhanced FOCA in cerebellum. It has been widely acknowledged that the cerebellum is crucial for motor control, and contributes to coordination instead of motion initiation. At the same time, the cerebellum integrates the information from many brain regions including sensory systems and spinal cord into fine motor activity (Fine et al., 2002). The abnormality in cerebellum might cause disturbance of movement, balance, and motor learning (Fine et al., 2002). Besides, the cerebellum also takes part in cognitive, affective, and sensory functions (Wolf et al., 2009). Traditionally, GTCS is viewed as a cortical phenomenon, but there were studies of patients with seizures showed seizures were originated in cerebellar gangliogliomas (Harvey et al., 1996), indicating that at least in cerebellar pathological conditions, seizures may indeed begin in the cerebellum (Norden and Blumenfeld, 2002). Here, the abnormal increased synchronous activity in cerebellum provides potential evidence to support that the cerebellum is a crucial region in GTCS.

## Conclusion

In this research, we used FOCA to study the pattern of local spontaneous activity in the epilepsy with GTCS only patients. Compared with healthy controls, patients with GTCS demonstrated higher spatiotemporal consistency in the motor-related cortex, and decreased FOCA in regions of DMN. Furthermore, the altered FOCA in several regions demonstrated significant correlation with the onset age and duration of epilepsy. These findings might reflect the disturbed baseline status of the brain and hyper-exciting spontaneous activity in motor-related regions in patients with GTCS. These results also provide evidence to support that the FOCA methods might be a potential tool to study intrinsic epileptic activity, which could help to understand the underlying pathophysiological mechanisms of GTCS.

## Ethics statement

This study was carried out in accordance with the recommendations of “Declaration of Helsinki” with written informed consent from all subjects. The protocol was approved by the ethical committee of the University of Electronic Science and Technology of China.

## Author contributions

SM, SJ, and CL wrote the paper. SJ, LY, and CL conceived the data analysis procedure. SM, HS, QZ, and LY collected the clinical data. RP, XJ, and JL recorded MRI data sets. SJ and RP performed the data analysis. IG provided some useful suggestions in paper writing.

### Conflict of interest statement

The authors declare that the research was conducted in the absence of any commercial or financial relationships that could be construed as a potential conflict of interest.
